# Associations between Japanese calligraphy practice and sleep quality in community-dwelling older adults: A cross-sectional Study

**DOI:** 10.1016/j.sleepx.2024.100124

**Published:** 2024-08-20

**Authors:** Georg von Fingerhut, Keitaro Makino, Osamu Katayama, Ryo Yamaguchi, Daiki Yamagiwa, Jessica K. Bone, Hiroyuki Shimada

**Affiliations:** a Department of Preventive Gerontology, Center for Gerontology and Social Science, National Center for Geriatrics and Gerontology, 7-430 Morioka-cho, Obu City, Aichi, 474-8511, Japan; b International Antique Institute, 1-8-39, Edogawa, Edogawa-ku, Tokyo, 132-0013, Japan; c Japan Society for the Promotion of Science, 5-3-1 Kojimachi, Chiyoda-ku, Tokyo, 102-0083, Japan; d Cognitive Neuroscience Division, Department of Neurology, Columbia University Irving Medical Center, 710 West 168th Street, New York, 10032, United States; e Research Department of Behavioural Science and Health, Institute of Epidemiology & Health Care, University College London, 1-19 Torrington Place, London, WC1E 7HB, United Kingdom

**Keywords:** Sleep, Calligraphy, Insomnia, Older adults, Art, Culture

## Abstract

**Background:**

Sleep disturbances, such as insomnia, are common among the elderly population and have been associated with negative health outcomes. Japanese calligraphy is a traditional art practice previously associated with various health benefits, such as stress reduction and improved cognitive function; however, its association with sleep quality has not been fully explored.

**Methods:**

This cross-sectional study included 21,207 subjects with basic attributes, health status, depressive symptoms, artistic practices, and sleep habits. Individuals who satisfied the chronic insomnia criteria were categorized into the following subtypes: sleep onset latency (SOL) insomnia, early morning awakening (EMA) insomnia, and wake after sleep offset (WASF) insomnia. The *t*-test, chi-square test, and logistic regression analysis were used to determine the association between Japanese calligraphy practice and sleep quality.

**Results:**

In this study, 17,597 elderly Japanese individuals were included, among whom 13.7 % practiced Japanese calligraphy. Regarding sleep characteristics, 32.0 % had chronic insomnia, 13.1 % had SOL insomnia, 9.1 % had EMA insomnia, and 14.2 % had WASF insomnia. Japanese calligraphy practice was associated with lower rates of chronic insomnia (odds ratio [OR] = 0.85, 95 % confidence interval (CI) = 0.76–0.95), including SOL insomnia (OR = 0.84, 95 % CI = 0.71–0.98), and EMA insomnia (OR = 0.80, 95 % CI = 0.66–0.97) but had no significant association with WASF insomnia.

**Conclusions:**

This study suggests that Japanese calligraphy practice is associated with lower odds of insomnia, particularly SOL and EMA insomnia. Calligraphy may be an effective nonpharmacological intervention for insomnia and poor sleep quality among elderly Japanese individuals.

## Highlights

• Japanese calligraphy linked to lower chronic, onset, and early morning insomnia.
• Haiku practice had no associations with sleep quality.
• Integrating calligraphy into therapies beneficial for sleep quality in older adults.

## Abbreviations:

CI: Confidence interval
EMA: Early morning awakening
GDS: Geriatric Depression Scale
MMSE: Mini-Mental State Examination
OR: Odds ratio
SOL: Sleep onset latency
WASF: Wake After Sleep Offset

## 1 Introduction

Sleep is a fundamental physiological process that plays a vital role in maintaining overall health and well-being. Insufficient or poor-quality sleep has been linked to several negative health outcomes, including an increased risk of chronic illnesses, cognitive decline, and mental health disorders [1]. Because of age-related sleep fragmentation, sleep onset and maintenance issues are common in older adults [2], whereas 30%–48 % of older adults’ experience insomnia symptoms [3]. Effective treatments for insomnia include behavioral, cognitive, and pharmacological interventions [4]; however, particularly in older individuals, sleeping pills have been associated with daytime drowsiness, increased risk of fall accidents during sleep [5], and negative effects on cognitive function [6]. Consequently, the combination of behavioral and cognitive therapies is considered an important nonpharmacological treatment to alleviate insomnia symptoms, particularly in older adults.

Recently, interest in exploring the potential benefits of various nonpharmacological activities on sleep quality and overall well-being among older adults has been growing. Cognitive behavioral therapy, a recommended treatment for insomnia, eliminates the risk of drug interactions, adverse events, and dependency [7]. Japanese calligraphy is a traditional form of artistic expression with deep cultural significance. Rooted in ancient Chinese calligraphy, Japanese calligraphy emerged as a distinctive art form during the Heian period (794–1185 AD) and has since evolved into a revered practice [8]. Japanese calligraphy involves the skilled and deliberate creation of characters using a brush and sumi ink on delicate rice paper, combining elements of esthetics, spirituality, and mindfulness [9]. As a therapeutic practice, calligraphy combines physical and mental coordination, visual interpretation, strategic planning through precise brushstrokes, and a focused mindset [10]. Wu et al. [11] highlighted the positive impact of calligraphy practice on emotional regulation and mental health in older adults. Calligraphy therapy has cognitive benefits, particularly in older individuals [12]. Hsiao et al. [13] recently showed that the daily practice of Chinese calligraphy handwriting could be an inexpensive and worthwhile way for older adults with mild cognitive impairment to improve cognition, psychological symptoms, and hand stability. Considering the cognitive demands of Japanese calligraphy and its stress reduction effects, it is reasonable to speculate that sleep quality may be influenced by overall engagement in this artistic practice. However, to date, no research has explored the association between engagement in Japanese calligraphy and sleep quality, insomnia, or other sleep characteristics.

This cross-sectional study explored the association between sleep and Japanese calligraphy practice among community-dwelling older adults.

## 2 Material and methods

### 2.1 Population and settings

**Fig. 1:**
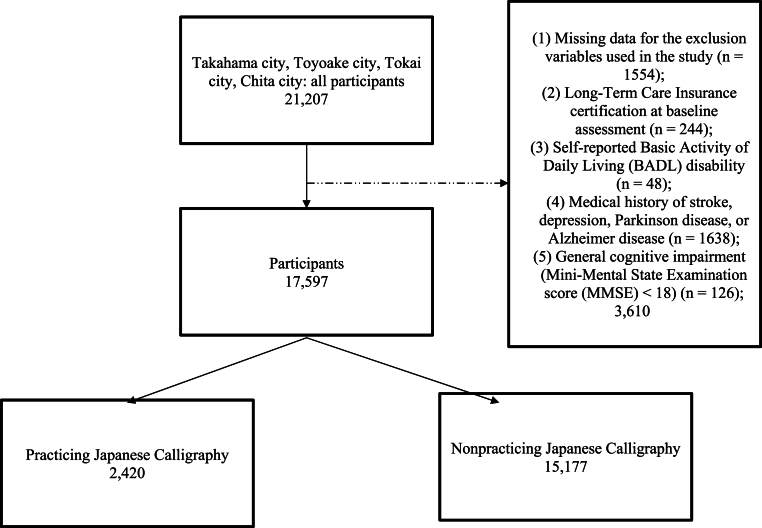
Flow diagram of patient inclusion.

This cross-sectional study comprised 21,207 community-dwelling older adults aged ≥65 years living in Takahama City (2015), Tokai City (2017), Toyoake City (2017), and Chita City (2019–2020) in Aichi Prefecture, Japan. In total, 3610 participants were excluded to form a healthy sample in line with previous research [14] based on the following criteria: (1) missing data for the exclusion variables used in the study (n = 1554); (2) Long-Term Care Insurance certification at baseline assessment (n = 244); (3) self-reported Basic Activity of Daily Living disability (n = 48); (4) medical history of stroke, depression, Parkinson's disease, and Alzheimer's disease (n = 1638); and (5) general cognitive impairment (Mini-Mental State Examination score [MMSE] < 18) (n = 126) because these conditions could influence sleep quality. In the analysis, 17,597 participants were finally included (Fig. 1).

The authors provided training to all personnel on the assessment protocols before the study started, and all baseline assessments were performed by qualified nurses and study assistants at community centers. This study adhered to the principles outlined in the Declaration of Helsinki, and participant data were used only once their consent was obtained. This study was approved by the Ethics Committee of the National Center for Geriatrics and Gerontology (1440–5).

### 2.2 Measures

#### 2.2.1 Outcomes

The participants were asked about their typical sleep and wake schedules. Each participant self-reported the time taken for sleep onset and offset during the previous month. Excessive daytime sleepiness was assessed using the question “How often do you have daytime sleepiness that requires a nap?” [14]. Personal dissatisfaction with sleep quality was assessed using the question “Are you satisfied with your sleep?” The following response options were used: “very satisfied,” “satisfied,” “dissatisfied,” and “very dissatisfied.” These responses were divided into two categories: “dissatisfied” and “satisfied.” Similarly, dissatisfaction with the sleep environment was assessed using the question “Are you satisfied with your sleeping environment, including light, room temperature, noise, and bedding (pillows)?” The responses were as follows: “very satisfied,” “satisfied,” “dissatisfied,” and “very dissatisfied.” These responses were divided into two categories: “dissatisfied” and “satisfied.” Further measurement of sleep habits in this study involved the assessment of the presence and frequency of specific symptoms, such as subjective sleep insufficiency; sleep-related impairment of motivation, concentration, and daytime functioning; delayed sleep phase; sleep-disturbing activities; and the use of sleeping pills. Each question had the following response options: “3 or more days a week,” “1 or 2 days a week,” “less than a day a week,” and “never.” These responses were divided into two categories: “3 or more days in a week” and “other.”

According to the Diagnostic and Statistical Manual of Mental Disorders (DSM-5) and the International Classification of Sleep Disorders (ICSD-3) criteria, chronic insomnia was characterized, which required experiencing at least one of the following issues: difficulty in falling asleep, difficulty initiating sleep, or early morning awakenings for ≥3 days per week; daytime sleepiness; and/or dissatisfaction with sleep occurring ≥3 days per week [15,16]. Participants who met the criteria for chronic insomnia were further divided into subtypes. Early morning awakening (EMA) insomnia was defined as early morning awakening after which participants could not sleep for 3 days per week or more during the last month [17]. Sleep onset latency (SOL) insomnia was defined as having experienced SOL of ≥30 min 3 days per week or more during the last month [18]. Wake after sleep offset (WASF) insomnia was defined as wakefulness ≥30 min after the offset of sleep for 3 days per week or more during the last month.

#### 2.2.2 Exposure

Japanese calligraphy practice was assessed using the question “Have you been engaged in Japanese calligraphy practice during the past year?” and the following options were used: “Yes” and “No.”

#### 2.2.3 Potential confounding factors

Sociodemographic (age, sex, academic background, and working status) and medical (weight (kg), height (m), and number of medications used) data were collected. The body mass index was computed as the weight in kilograms divided by the square of the height in meters. Eye diseases were evaluated by considering the presence of conditions, such as glaucoma, cataract, or other eye diseases. In face-to-face interviews, the participants were asked about their medical conditions (diabetes mellitus, respiratory disease, urination issues, heart disease, hypertension, and hyperlipidemia) and alcohol consumption status. Depressive symptoms were measured using the 15-item Geriatric Depression Scale (GDS) (range: 0–15), with higher scores indicating more depressive symptoms, whereas scores of ≥6 were identified as having significant depressive symptoms [19]. The MMSE was used to assess global cognitive function [20]. Physical activity levels were determined based on the participants’ self-reported engagement in physical activity: (i) “Do you engage in moderate levels of physical exercise or sports aimed at health?” and (ii) “Do you engage in low levels of physical exercise aimed at health?” [21].

Haiku practice was used as a confounding factor for another type of artistic activity. Haiku is another form of Japanese art that is less common than calligraphy. It is characterized by its brevity, focuses on capturing a moment or experience in nature, and is classified as written poetry. It usually comprises three lines following a syllable pattern of 5–7–5, and similar to calligraphy as an artistic practice, it is physically reflected in the depiction of Japanese kanji [22]. Haiku practice was assessed using the question “Have you been engaged in Haiku practice during the past year?” and the following options were used: “Yes” and “No.”

### 2.3 Statistical analysis

The participants' baseline characteristics were initially investigated according to whether they engaged in Japanese calligraphy. Which aspects of sleep were associated with Japanese calligraphy practice was then explored using Pearson's chi-square test for categorical variables and Student's t-test for continuous variables. When there was evidence of an association between calligraphy engagement and sleep characteristics, we further explored whether this association was attenuated after adjusting for confounders in logistic regression models. The results were adjusted for confounding factors, including age, sex, academic background, MMSE score, depressive symptoms, alcohol and medication use, light and regular exercise, heart disease, respiratory disease, eye illnesses, diabetes mellitus, hypertension, hyperlipidemia, urination issues, and time in bed. First, the association between Japanese calligraphy and insomnia was tested. Furthermore, a mediation analysis was conducted using the Hayes AF. PROCESS macro for SPSS version 4.2 (available from: https://www.processmacro.org/download.html) to examine whether depression (GDS score) mediates the relationship between calligraphy practice and insomnia. The analysis controlled for age, sex, academic background, MMSE score, alcohol and medication use, light and regular exercise, heart disease, respiratory disease, eye illnesses, diabetes mellitus, hypertension, hyperlipidemia, urination issues, and time in bed. Model 4, with bias-corrected bootstrap sampling (10,000 samples) was used to estimate the 95 % confidence intervals (CIs) for the indirect effect. Coefficients for paths to insomnia (binary outcome) are expressed in log-odds units. The association of Japanese calligraphy with each insomnia subtype was then explored separately. Odds ratios (ORs) and 95 % CIs were estimated, with the statistical significance level set at *p* < 0.05 for all analyses. All analyses were performed using Statistical Package for the Social Sciences, version 25 (IBM Corp, Armonk, NY, USA).

## 3 Results

In this study, 17,597 older adults (mean age, 73.9 ± 5.7 years; 7905 (44.9 %) males; 11.8 ± 2.4 years of academic background) were included. Among them, 2420 participants (13.7 %) practiced Japanese calligraphy. Table 1 shows the baseline characteristics of the participants according to whether they practiced calligraphy. Those who practiced calligraphy were on average older (74.5 *vs.* 73.8 years), more likely to be female (67.6 % *vs.* 53.1 %), and used fewer medications. Participants who practiced calligraphy had a higher mean MMSE score and were less likely to have depressive symptoms (GDS score ≥6). Statistically significant differences in the performance of light (*p* < 0.001) and regular (*p* < 0.001) exercises were observed between the two groups. Among the various health conditions listed, only the percentage of participants with hypertension significantly differed between the two groups (*p* < 0.001).

**Table 1:** Participants characteristics (N = 17,597).

		Total N = 17,597	Practicing Japanese calligraphy n = 2420	Nonpracticing Japanese calligraphy n = 15,177	*p*
Age (years)		73.9 ± 5.7	74.5 ± 5.7	73.8 ± 5.7	<0.001*^a^
Sex (%)	Female	9692 (55.1)	1637 (67.6)	8055 (53.1)	<0.001*
	Male	7905 (44.9)	783 (32.4)	7122 (46.9)	
Academic Background (years)		11.8 ± 2.4	12.2 ± 2.3	11.7 ± 2.4	<0.001*^a^
Work (%)	No	12541 (71.3)	1757 (72.6)	10784 (71.0)	0.122
	Yes	5056 (28.7)	663 (27.4)	4393 (29.0)	
Alcohol Use (%)	No	10473 (59.5)	1499 (61.9)	8974 (59.1)	0.009*
	Yes	7124 (40.5)	921 (38.1)	6203 (40.9)	
Number of Medication Used (number)		3.0 ± 2.8	2.9 ± 2.8	3.1 ± 2.8	0.006*^a^
MMSE Score		27.1 ± 2.5	27.2 ± 2.5	27.0 ± 2.5	<0.001*^a^
Depressive symptoms (GDS score)	<6	14413 (81.9)	2141 (88.5)	12272 (80.9)	<0.001*
	≥6	3184 (18.1)	279 (11.5)	2905 (19.1)	
BMI (kg/m2)	<25	12772 (72.6)	1771 (73.2)	11001 (72.5)	0.492
	≥25	4825 (27.4)	649 (26.8)	4176 (27.5)	
Light Exercise (days per week) (%)	7	6133 (34.8)	975 (40.3)	5158 (34.0)	<0.001*
	5–6	1244 (7.1)	160 (6.6)	1084 (7.1)	
	2–4	3782 (21.5)	590 (24.4)	3192 (21.0)	
	<1	1438 (8.2)	196 (8.1)	1242 (8.2)	
	0	5000 (28.4)	499 (20.6)	4501 (29.7)	
Regular Exercise (days per week) (%)	7	493 (2.8)	88 (3.6)	405 (2.7)	<0.001*
	5–6	625 (3.6)	84 (3.5)	541 (3.6)	
	2–4	3323 (18.9)	558 (23.1)	2765 (18.2)	
	<1	2280 (12.9)	387 (16.0)	1893 (12.5)	
	0	10876 (61.8)	1303 (53.8)	9573 (63.0)	
Haiku Practice (%)	No	16326 (92.8)	1905(78.7)	14421(95.0)	<0.001*
	Yes	1271 (7.2)	515 (21.3)	756 (5.0)	
Heart Diseases (%)	No	14627 (83.1)	1979 (81.8)	12648 (83.3)	0.057
	Yes	2970 (16.9)	441 (18.2)	2529 (16.7)	
Respiratory Diseases (%)	No	15576 (88.5)	2152 (88.9)	13424 (88.4)	0.513
	Yes	2021 (11.5)	268 (11.1)	1753 (11.6)	
Eye Illnesses (%)	No	8744 (49.7)	1207 (49.9)	7537 (49.7)	0.861
	Yes	8853 (50.3)	1213 (50.1)	7640 (50.3)	
Diabetes (%)	No	15165 (86.2)	2088 (86.3)	13077 (86.2)	0.897
	Yes	2432 (13.8)	332 (13.7)	2100 (13.8)	
Hypertension (%)	No	9307 (52.9)	1356 (56.0)	7951 (52.4)	0.001*
	Yes	8290 (47.1)	1064 (44.0)	7226 (47.6)	
Hyperlipidaemia (%)	No	11223 (63.8)	1565 (64.7)	9658 (63.6)	0.328
	Yes	6374 (36.2)	855 (35.3)	5519 (36.4)	
Urination Issues (%)	No	10268 (58.3)	1414 (58.4)	8854 (58.3)	0.947
	Yes	7329 (41.7)	1006 (41.6)	6323 (41.7)	

*Note*: *p < 0.05, mean ± SD χ^2^ test, ^a^Student's T-test, BMI = body mass index, GDS = Geriatric Depression Scale, MMSE = Mini-Mental State Examination.

Regarding sleep characteristics, 5638 participants (32.0 %) had chronic insomnia, 1600 (9.1 %) had EMA insomnia, 2312 (13.1 %) had SOL insomnia, and 2499 (14.2 %) had WASF insomnia (Table 2). The mean time spent in bed was 462.8 min for the entire study population. Participants who practiced Japanese calligraphy spent an average of 459.0 min in bed, which was shorter than that in those who did not practice Japanese calligraphy (463.4 min) (*p* = 0.008). Among participants who practiced Japanese calligraphy, 689 (28.5 %) reported chronic insomnia, and 168 (6.9 %) reported EMA insomnia, showing a statistically significant difference between the two groups (*p* < 0.001). The percentage of participants with SOL insomnia was lower among those who practiced Japanese calligraphy (11.3 %) than among those who did not practice Japanese calligraphy (13.4 %), showing a statistically significant difference (*p* = 0.005). The percentage of participants with WASF insomnia was not significantly different (*p* = 0.273) between the two groups. For the remaining sleep-related factors, no significant differences were observed between participants who practiced Japanese calligraphy and those who did not.

**Table 2:** Participants sleep characteristics (N = 17,597).

		Total, N = 17,597	Practicing Japanese Calligraphy, n = 2420	Nonpracticing Japanese Calligraphy, n = 15,177	*p*
Time in Bed (min)		462.8 ± 76.1	459.0 ± 75.2	463.4 ± 76.3	0.008*^a^
Subjective Sleep Insufficiency (times a week) (%)	<3	16903 (96.1)	2342 (96.8)	14561 (95.9)	0.051
	≥3	694 (3.9)	78 (3.2)	616 (4.1)	
Excessive Daytime Sleepiness (times a week) (%)	<3	16894 (96.0)	2339 (96.6)	14555 (95.9)	0.095
	≥3	699 (4.0)	81 (3.4)	618 (4.1)	
Sleep-Related Impairment of Motivation, Concentration, and Daytime Functioning (times a week) (%)	<3	17476 (99.3)	2407 (99.5)	15069 (99.3)	0.494
	≥3	116 (0.7)	13 (0.5)	103 (0.7)	
Sleeping Pills use (times a week) (%)	<3	16537 (94.0)	2270 (93.8)	14267 (94.0)	0.716
	≥3	1060 (6.0)	150 (6.2)	910 (6.0)	
Presence of Sleep-Disturbing Activities (times a week) (%)	<3	17380 (98.8)	2392 (98.8)	14988 (98.8)	0.911
	≥3	210 (1.2)	28 (1.2)	182 (1.2)	
Delayed Sleep Phase (times a week) (%)	<3	17453 (99.2)	2404 (99.3)	15049 (99.2)	0.532
	≥3	140 (0.8)	16 (0.7)	124 (0.8)	
Sleep Quality Dissatisfaction (%)	No	16943 (96.3)	2339 (96.6)	14604 (96.2)	0.353
	Yes	650 (3.7)	81 (3.4)	569 (3.8)	
Sleep Environment Dissatisfaction (%)	No	17522 (99.6)	2409 (99.5)	15113 (99.6)	0.602
	Yes	70 (0.4)	11 (0.5)	59 (0.4)	
Chronic Insomnia (%)	No	11959 (68.0)	1731 (71.5)	10228 (67.4)	<0.001*
	Yes	5638 (32.0)	689 (28.5)	4949 (32.6)	
Sleep Onset Insomnia (%)	No	15285 (86.9)	2146 (88.7)	13139 (86.6)	0.005*
	Yes	2312 (13.1)	274 (11.3)	2038 (13.4)	
Early Morning Awakening Insomnia (%)	No	15997 (90.9)	2252 (93.1)	13744 (90.6)	<0.001*
	Yes	1600 (9.1)	168 (6.9)	1432 (9.4)	
Wake after Sleep Offset Insomnia (%)	No	15098 (85.8)	2094 (86.5)	13004 (85.7)	0.273
	Yes	2499 (14.2)	326 (13.5)	2173 (14.3)	

*Note*: *p < 0.05, χ^2^ test, ^a^Student's T-test, min = minutes.

**Fig. 2:**
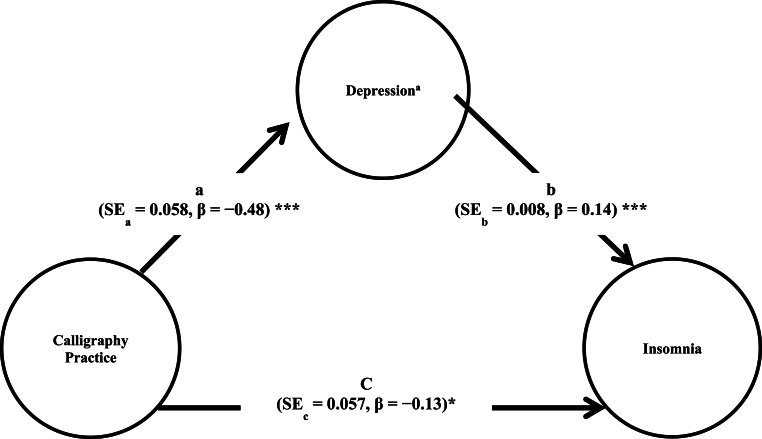
Mediation model of depression and the relationship between calligraphy practice and insomnia. Note: significance level of the unstandardized path coefficients (***p < 0.01, *p < 0.05), SE = Standard Error. ^a^A higher score on the scale indicates higher score for depression.

The logistic regression analysis results showed that practicing Japanese calligraphy was associated with a lower odds of experiencing chronic insomnia in the crude model (OR = 0.82, 95 % CI = 0.75–0.90) and model 1 adjusted by Haiku practice (OR = 0.84, 95 % CI = 0.76–0.93) (Table 3). The association remained even after controlling for potential confounding factors (OR = 0.85, 95 % CI = 0.76–0.95). In addition, the results of mediation analysis indicated a significant direct effect of calligraphy practice on insomnia risk (β = −0.135, Standard Error [SE] = 0.057, Z = −2.371, p = 0.018, 95 % CI [−0.247, −0.023]). The indirect effect through depression symptoms was also significant (β = −0.069, Boot SE = 0.008, 95 % Boot CI [−0.086, −0.053]), suggesting partial mediation. Calligraphy practice was associated with lower depression scores (β = −0.481, SE = 0.058, p < 0.001), which in turn were associated with lower insomnia risk (β = 0.143, SE = 0.008, p < 0.001). The total effect model explained 6.69 % of the variance in insomnia risk (McFadden R^2^ = 0.0669) (Fig. 2).

**Table 3:** Associations between chronic insomnia and practice of Japanese calligraphy by logistic regression models (N = 17,597).

	Crude Model		Model 1		Model 2	
Variable	OR (95 % CI)	*p*	OR (95 % CI)	*p*	OR (95 % CI)	*p*
Practice of Japanese calligraphy (No = 0, Yes = 1)	0.82 (0.75–0.90)	<0.001*	0.84 (0.75–0.93)	0.001*	0.85 (0.76–0.95)	0.004*
Haiku Practice (No = 0, Yes = 1)			1.01 (0.88–1.15)	0.900	1.03 (0.90–1.18)	0.683
Gender (F = 0, M = 1)					0.74 (0.680–0.81)	<0.001*
Age (years)					1.02 (1.01–1.02)	<0.001*
Education (years)					0.98 (0.96–0.99)	0.009
MMSE (score)					1.03 (1.01–1.05)	<0.001*
Depressive Symptoms (GDS <6 = 0, ≥6 = 1)					2.07 (1.88–2.27)	<0.001*
Alcohol Use (No = 0, Yes = 1)					1.02 (0.94–1.10)	0.691
Number of Medication (number)					1.02 (1.01–1.04)	0.003*
Light Exercise (days per week)					0.98 (0.96–1.01)	0.169
Regular Exercise (days per week)					0.98 (0.95–1.02)	0.304
Heart Disease (No = 0, Yes = 1)					1.07 (0.97–1.18)	0.185
Respiratory Disease (No = 0, Yes = 1)					1.09 (0.98–1.22)	0.118
Eyes Illnesses (No = 0, Yes = 1)					1.11 (1.03–1.21)	0.007*
Diabetes (No = 0, Yes = 1)					0.99 (0.89–1.11)	0.962
Hypertension (No = 0, Yes = 1)					0.94 (0.87–1.02)	0.139
Hyperlipidaemia (No = 0, Yes = 1)					1.06 (0.98–1.15)	0.125
Urination Issues (No = 0, Yes = 1)					1.11 (1.02–1.20)	0.010*
Time In Bed Time (min)					1.01 (1.00–1.01)	<0.001*

*Not*e: *p < 0.05, MMSE = Mini-Mental State Examination, GDS = Geriatric Depression Scale, CI = confidence interval.

Furthermore, practicing Japanese calligraphy was statistically significantly associated with a lower risk of EMA insomnia in the crude (OR = 0.7, 95 % CI = 0.61–0.84) and adjusted models (OR = 0.80, 95 % CI = 0.66–0.97) and a lower risk of SOL insomnia in the crude (OR = 0.8, 95 % CI = 0.72–0.94) and adjusted models (OR = 0.84, 95 % CI = 0.71–0.98). In contrast, practicing Japanese calligraphy was not significantly associated with WASF insomnia in the crude (*p* = 0.27) or adjusted (*p* = 0.34) models (Table 4).

**Table 4:** Associations between insomnia subtypes and practice of Japanese calligraphy by logistic regression models (N = 17,597).

	Sleep Onset Insomnia		Early Morning Awakening Insomnia		Wake after Sleep Offset Insomnia	
	Adjusted Model		Adjusted Model		Adjusted Model	
Variable	OR (95 % CI)	*p*	OR (95 % CI)	*p*	OR (95 % CI)	*p*
Practice of Japanese Calligraphy (No = 0, Yes = 1)	0.84 (0.71–0.98)	0.026*	0.80 (0.66–0.97)	0.021*	0.93 (0.80–1.08)	0.335
Haiku Practice (No = 0, Yes = 1)	1.19 (0.99–1.42)	0.064	0.92 (0.73–1.16)	0.497	0.90 (0.75–1.08)	0.266
Gender (F = 0, M = 1)	0.61 (0.54–0.69)	<0.001*	0.82 (0.71–0.94)	0.005*	0.65 (0.58–0.73)	<0.001*
Age (years)	0.99 (0.99–1.01)	0.811	1.02 (1.01–1.03)	0.001*	1.02(1.01–1.03)	<0.001*
Education (years)	0.99 (0.96–1.01)	0.310	0.94 (0.92–0.97)	<0.001*	0.98 (0.96–1.00)	0.126
MMSE (score)	1.01 (0.99–1.03)	0.462	0.99 (0.97–1.01)	0.428	1.07 (1.05–1.09)	<0.001*
Depressive Symptoms (GDS <6 = 0, ≥6 = 1)	1.73 (1.54–1.96)	<0.001*	2.29 (2.01–2.61)	<0.001*	1.34 (1.19–1.51)	<0.001*
Alcohol Use (No = 0, Yes = 1)	1.00 (0.89–1.12)	0.999	1.10 (0.97–1.26)	0.137	1.04 (0.93–1.16)	0.460
Number of Medication (number)	1.03 (1.01–1.05)	0.011*	1.01 (0.98–1.03)	0.549	1.00 (0.98–1.02)	0.900
Light Exercise (days per week)	0.96 (0.93–0.99)	0.015*	0.99 (0.96–1.03)	0.903	1.00 (0.97–1.03)	0.781
Regular Exercise (days per week)	0.98 (0.93–1.03)	0.339	0.95 (0.89–1.01)	0.086	1.01 (0.97–1.06)	0.529
Heart Disease (No = 0, Yes = 1)	1.06 (0.92–1.22)	0.394	1.06 (0.90–1.24)	0.494	1.11 (0.98–1.27)	0.107
Respiratory Disease (No = 0, Yes = 1)	0.96 (0.82–1.13)	0.644	1.21 (1.02–1.43)	0.031*	1.06 (0.92–1.23)	0.412
Eyes Illnesses (No = 0, Yes = 1)	1.01 (0.90–1.12)	0.980	1.23 (1.08–1.40)	0.002*	1.08 (0.98–1.20)	0.123
Diabetes (No = 0, Yes = 1)	1.04 (0.89–1.21)	0.622	0.97 (0.81–1.16)	0.744	1.07 (0.93–1.24)	0.355
Hypertension (No = 0, Yes = 1)	0.95 (0.85–1.06)	0.324	0.98 (0.86–1.11)	0.734	0.96 (0.86–1.06)	0.413
Hyperlipidaemia (No = 0, Yes = 1)	1.168 (1.048–1.303)	0.005*	1.05 (0.92–1.19)	0.442	1.01 (0.91–1.12)	0.824
Urination Issues (No = 0, Yes = 1)	0.99 (0.89–1.10)	0.848	1.10 (0.97–1.24)	0.143	1.01 (0.92–1.12)	0.776
Time In Bed Time (min)	1.01 (1.01–1.01)	<0.001*	1.00 (1.00–1.00)	<0.001*	1.01 (1.01–1.01)	<0.001*

*Not*e: *p < 0.05, MMSE = Mini-Mental State Examination, GDS = Geriatric Depression Scale, CI = confidence interval.

## 4 Discussion

This study examined the association between Japanese calligraphy practice and sleep quality among older Japanese individuals. This study revealed that older Japanese adults who practiced Japanese calligraphy reported better sleep quality, as indicated by the lower rates of chronic insomnia, including EMA and SOL insomnia.

### 4.1 Associations between Japanese calligraphy practice and insomnia

To the best of our knowledge, this is the first study to show the association between Japanese calligraphy practice and sleep quality, particularly insomnia subtypes, among a Japanese community-dwelling older population. Two studies have reported an association between calligraphy and insomnia. In a randomized controlled trial involving patients with nasopharyngeal cancer, Yang et al. [23] compared the effects of Chinese calligraphy handwriting with muscle relaxation and imagery training in 79 patients with cancer (aged 22–71 years). Both calligraphy and relaxation training were effective in relieving mood disturbances and insomnia symptoms as measured by the Symptom Distress Scale, and calligraphy also increased concentration levels [23]. In another randomized controlled trial by Fung et al. [24], 90 patients (age range: 16–70 years) with primary insomnia were assigned to receive 8 weeks of Chinese Guqin music, Chinese calligraphy handwriting, or control rest sessions. They found that calligraphy practice increased frontal delta, theta, and alpha waves on electroencephalogram, indicating enhanced relaxation, attention, comprehension, creativity, and psychological recovery [24]. Therefore, they concluded that calligraphy and Guqin music positively impact cardiac and brain function, demonstrating their efficacy as interventions for insomnia through mind–body regulation [24]. These results align with those of this study, which showed that Japanese calligraphy is associated with lower insomnia rates among community-dwelling older Japanese adults.

Studies have suggested that several mechanisms underlie the association between calligraphy and improved sleep. Calligraphy practice is associated with muscle and emotional relaxation and involves creating a calm and organized space that relaxes the body. It requires assuming an upright posture, holding the brush correctly at a specific angle, lifting it gently while inhaling, and pressing it onto the paper while exhaling [10,13]. It has been linked to reduced stress and improved emotional well-being, mindfulness, and calmness [11,25,26]—psychological states that could promote healthy sleep. Thus, many participants might associate the act of practicing calligraphy with a sense of relaxation and stress relief, which, in turn, appeared to positively affect their sleep quality. Moreover, Chu et al. [27] conducted a systematic review and meta-analysis showing that Chinese calligraphy therapy significantly reduces the neuropsychiatric symptoms of depression. Because calligraphy practice requires regulated breath control and a focused mindset, it has also been linked to slower respiration rates and decreased heart rate and blood pressure levels [28]. Together with the meditative nature of calligraphy, which requires mindful focus and rhythmic brushstrokes, it may induce relaxation and emotional regulation that buffers against depressive symptoms [26]. In fact, our mediation analysis showed depression partially mediates the association between calligraphy practice and insomnia. Calligraphy linked to lower depression scores and reduced insomnia likelihood, with both direct and indirect effects on sleep. This finding aligns with previous research demonstrated that depression can mediate the relationship between social participation and health outcomes, including sleep disorders [29]. Nevertheless, calligraphy practice maintained a direct effect on insomnia after accounting for depression, and the model's modest explanatory power (6.69 % of variance) indicates additional pathways through which it may improve sleep. For instance, mastering calligraphy techniques and appreciating the esthetic quality of finished works can provide a sense of accomplishment and satisfaction that improves mood [13]. Social engagement with other calligraphy practitioners may also enhance well-being and reduce the isolation linked to depression [10]. Considering these multifaceted psychological and social mechanisms, calligraphy has significant potential as an effective intervention for reducing depressive symptoms and improving sleep quality in older adults.

Recent neuroimaging studies have revealed that calligraphy practice reshapes the posterior cingulate cortex, which is a brain region associated with attention and self-referential processing [30]. The posterior cingulate cortex is active when individuals are not focused on the external environment [31]. Dysfunction of this network has been implicated in various mental health conditions, including depression [32] and insomnia [33]. Chronic sleep disturbance has been linked to increased cortical atrophy and volume loss in the hippocampus and posterior cingulate cortex in cognitively healthy older adults [34]. These findings highlight the importance of understanding the effects of calligraphy practice and sleep quality on brain regions involved in attention, self-referential processing, and cognitive functioning. All aforementioned findings could explain the physiological association between calligraphy practice and insomnia; however, the mechanisms underlying the connection between calligraphy and sleep remain unclear.

Note that Haiku practice, which was used as a confounding factor for another type of artistic activity, showed no significant association with any type of insomnia. Similar to Japanese calligraphy, Haiku involves writing kanji characters and is recognized for its potential therapeutic benefits, such as stress reduction, increased mindfulness, and improved emotional well-being [22], empathy, and cultural well-being [35]. Thus, the potential positive association between Japanese calligraphy and insomnia may extend beyond relaxation effects. In fact, insomnia has been identified as a risk factor for cognitive decline and decreased attention [1]. Because each brush stroke in Japanese calligraphy is executed in a precise order, the requirement for focused attention and sequenced fine motor movements in calligraphy may improve cognitive function through positive effects on visual attention, behavioral changes, and cognitive activation [10]. Enhancing these cognitive functions may further improve sleep quality, creating a positive feedback loop. Although the benefits of calligraphy on cognition are well documented, the direct effects of Haiku practice on health remain unexplored, necessitating further research to understand this relationship. To the best of our knowledge, this is the first study to report the association between Haiku practice and sleep.

### 4.2 Associations between Japanese calligraphy practice and insomnia subtypes

In this study, insomnia was divided into subtypes based on their time-related association with the main sleep period. Our results suggest that insomnia is a heterogeneous process, with variations among its subtypes. In this study, older Japanese calligraphy practitioners were associated with significantly lower rates of both EMA insomnia and SOL insomnia than nonpractitioners; however, they had no significant associations with WASF insomnia.

SOL insomnia and EMA insomnia are common sleep disruptions in the elderly and are characterized by difficulty maintaining sleep, waking up earlier than desired, and inability to fall back asleep [2]. Among the subtypes of insomnia, those with sleep onset and maintenance issues tend to have greater mood disturbances than those with sleep offset issues [36]. This may be attributed to anxiety characteristics, which involve persistent worrying in the evening and upon waking in the morning, both of which can disrupt the onset of sleep. Conversely, by enhancing emotional regulation and alleviating anxiety and depression, the practice of calligraphy could mitigate mood disturbances that disrupt sleep maintenance [25,29]. The relaxing and meditative nature of calligraphy may help decrease cognitive and somatic arousal, which delays sleep onset and leads to EMAs. However, the specific mechanisms associated with calligraphy being able to improve SOL and EMA insomnia requires further elucidation using polysomnography, sleep diaries, and other methodologies.

Interestingly, in this study, no significant association was found between calligraphy and reduced WASF insomnia, which involves morning time spent in bed after sleep offset. To the best of our knowledge, this is the first study to examine the association between art practice and sleep offset parameters. Relaxation-based treatments are more effective in improving sleep onset rather than sleep offset parameters [37]. This finding is in accordance with the results of the current study. However, currently, there are no recommendations on the WASF insomnia cutoff point [38]. In this study, the cutoff point for WASF insomnia was based on that for SOL insomnia. Therefore, it might be important to consider another cutoff point for WASF insomnia.

Overall, calligraphy appears beneficial for alleviating EMA and SOL insomnia; however, its effects on WASF insomnia merit further investigation. Elucidating the mechanisms linking calligraphy to various insomnia subtypes can facilitate targeted behavioral sleep interventions.

### 4.3 Limitations

First, this study used cross-sectional data. Thus, its ability to determine causal relationships and focus on the practice of Japanese calligraphy might have been limited. The specific focus on Japanese calligraphy in this study can limit direct comparisons with studies on other forms of art, including Chinese calligraphy. Furthermore, it is important to note that all data in this study, including the primary outcomes on sleep, were self-reported, which may introduce reporting biases.

Second, this study did not provide detailed information about the timing, duration, frequency, and intensity of Japanese calligraphy practice. Studies have reported that 31 % of patients practice Chinese calligraphy before bedtime [23], whereas studies on the impact of visual art-making on cortisol levels showed that in 75 % of the participants, cortisol levels decreased during their 45 min of art-making, regardless of their past art experiences [39]. Nevertheless, some studies have suggested that regular and frequent practice is necessary to elicit significant sleep improvements, whereas others have proposed that even occasional practice can have benefits [40]. Because no information about Japanese calligraphy and Haiku practices among the Japanese population has been reported, the art practices were assessed during the past year. This definition helped include rare participants, whereas a large sample size could be a good representation of the population. The optimal timing, duration, and frequency of Japanese calligraphy practice for improving sleep quality requires further investigation.

## 5 Conclusion

In conclusion, this study highlights a positive association between Japanese calligraphy practice and lower risk of insomnia, particularly EMA and SOL insomnia. Considering the stress-reducing and cognitive demands of Japanese calligraphy and its relaxation effects, it may contribute to improved sleep quality. Further research is required to establish causality, explore psychosocial mechanisms, and generalize these findings to broader populations and cultural contexts. These findings contribute to the growing body of knowledge on the relationship between artistic practices and health among community-dwelling older adults and offer potential therapeutic implications for art-based interventions in sleep therapy.

## Funding

This study received support from following grants: JSPS KAKENHI (23K19791), the Research Funding for Longevity Sciences from the 10.13039/501100007312National Center for Geriatrics and Gerontology (29–42, 29–31) and 10.13039/100009619AMED (15dk0107003h0003, 15dk0207004h0203, 18le0110004h0002, 18dk0207027h0003, 19de0107002h0001). The funding sources did not influence the study's design, data gathering, analysis, interpretation, report composition, or the choice to publish the article. Industry sources did not provide any form of support.

## Data availability

In adherence to institutional guidelines and to ensure the privacy of participants, the complete dataset is not accessible to other researchers. The study described in the manuscript was not registered in advance.

## CRediT authorship contribution statement

**Georg von Fingerhut:** Writing – original draft, Formal analysis, Conceptualization. **Keitaro Makino:** Supervision, Data curation. **Osamu Katayama:** Data curation. **Ryo Yamaguchi:** Methodology. **Daiki Yamagiwa:** Methodology. **Jessica K. Bone:** Writing – review & editing, Methodology. **Hiroyuki Shimada:** Supervision.

## Declaration of competing interest

The authors declare that they have no known competing financial interests or personal relationships that could have appeared to influence the work reported in this paper.
